# The background and experience of neuroscience teachers in Australian universities: A cross-sectional survey

**DOI:** 10.1371/journal.pone.0311252

**Published:** 2024-10-02

**Authors:** Gabrielle Todd, Fabian Kreilaus, Amy S. Lonergan, Melissa S. Cameron, Kay L. Double

**Affiliations:** 1 UniSA Clinical & Health Sciences and Alliance for Research in Exercise, Nutrition and Activity (ARENA), University of South Australia, Adelaide, SA, Australia; 2 Brain and Mind Centre and School of Medical Sciences (Neuroscience), The University of Sydney, Sydney, NSW, Australia; 3 School of Medical Sciences (Education Innovation), The University of Sydney, Sydney, NSW, Australia; Far Eastern University - Manila, PHILIPPINES

## Abstract

Neuroscience is an academic discipline taught within a broad range of university degrees and programs. The expertise and experience of neuroscience teaching staff contribute to the student’s learning experience and knowledge acquisition. We undertook a survey to characterize the neuroscience teaching workforce and practices in Australian universities, and to investigate access to and deficiencies in neuroscience teaching resources and training. Two hundred neuroscience teaching staff completed our anonymous online survey. The responses indicated that neuroscience is primarily being delivered by highly qualified (86% with doctoral degrees, 27% with formal qualifications in tertiary teaching), research-active (45% were recently primary supervisors of research students) teaching academics with secure employment (77% in full-time continuing positions). There were more females (61.5%) than males (38.5%) in the sample and most respondents taught neuroscience to students enrolled in a range of degrees/programs. Most survey respondents could access an anatomy laboratory for teaching (87%) but access to specialised online resources, such as augmented reality presentations, customised game-based learning approaches, and/or online brain atlases, was limited. Most survey respondents reported they would benefit from increased access to neuroscience teaching resources and/or peer-tested teaching materials (80%), an informal network of Australian neuroscience teaching peers (64%), and/or training workshops on neuroscience teaching (59%). Approximately half of survey respondents supported the creation of national guidelines for neuroscience teaching curricula. The survey results identify specific gaps in teacher training and resources and inform the development of strategies to support tertiary teachers of neuroscience and student learning.

## Introduction

University academics typically perform one or more of the following categories of professional tasks: teaching, research, and/or service to the institution, community, and/or discipline [1, 2]. Expertise in one or more of these domains is typically acquired through participation in related practices deemed to be important by the profession and society [3]. The current study focuses on the expertise of tertiary teachers of neuroscience.

Neuroscience is a specialised discipline within anatomy and physiology concerned with the structure and function of the nervous system. Neuroscience includes a wide range of subspecialities including neuroanatomy, neuroimaging, and clinical, cognitive, and molecular neuroscience. Many tertiary-qualified professions require knowledge of the nervous system; medical clinicians, nurses, and allied health professionals, for example, apply neuroscience knowledge to the diagnosis, treatment, and rehabilitation of neurological diseases and disorders [1, 2]. Students pursuing a broad range of degrees are thus required or may elect to study university-level neuroscience. Neuroscience also informs formal learning strategies, including those used in tertiary education institutions [e.g. 3, 4].

University students may complete courses or units of study that focus specifically on the nervous system, or complete courses that include some neuroscience within the study of multiple organ systems and molecular pathways, at either the undergraduate or postgraduate levels. The quality of teaching provided in these courses plays an important role in determining the breadth and depth of the neuroscience knowledge of students, shaping the students learning experience and outcomes, and inspiring students to consider careers involving more advanced knowledge of the nervous system. Examples of factors that contribute to teaching quality are the expertise, experience, and professional development of the teaching staff. Expertise includes knowledge of discipline content (e.g., neuroscience content), pedagogical and curricular knowledge, and knowledge of discipline-specific teaching and learning strategies [1]. Shulman (1986) clearly articulated the importance of this knowledge in an article that accompanied their Presidential Address at the annual meeting of the American Educational Research Association: “*Teachers must not only be capable of defining for students the accepted truths in a domain*. *They must also be able to explain why a particular proposition is deemed warranted*, *why it is worth knowing*, *and how it relates to other propositions*, *both within the discipline and without*, *both in theory and in practice*” (page 6) [4]. Teacher expertise, experience, and professional development have a significant effect on student learning outcomes (e.g., [5]).

Whether neuroscience is taught by academic staff with expertise in the discipline of neuroscience, and appropriate training and experience in tertiary education, has not been assessed, nor the availability and quality of traditional teaching resources, such as anatomy laboratories and neuroanatomic models, and more recent online developments in education (e.g., interactive three-dimensional anatomic atlases) for neuroscience teaching is unknown. This gap in knowledge was identified through a systematic search of multiple major English-language journal databases (Medline, EMBASE, Emcare, ERIC, Scopus) using the search terms: “neuroscience” and “teach*” and “tertiary” and “university” (limited to English language and humans). Screening of the titles of resultant journal articles (n = 40 Medline, n = 1 EMBASE, n = 16 Emcare, n = 0 ERIC, n = 0 Scopus) yielded no primary research on the characteristics (demographics, qualifications, experience, training, or access to resources) of the tertiary neuroscience teaching workforce. Thus, we conducted a primary research descriptive study to address these knowledge gaps. The aim of our study was to characterize the neuroscience teaching workforce in Australian universities, and to investigate access to and gaps in neuroscience teaching resources and training. This topic is challenging to investigate across country borders due to differences in employment and professional accreditation standards, societal and cultural norms, and university and curriculum structure. Thus, we chose to focus on the Australian tertiary education sector because there are a large number of universities (39 public and 4 private) in Australia, and Australian universities must operate and deliver a standard of tertiary education that is compliant with the Australia Government Tertiary Education Quality and Standards Agency. The current study is the first description of a nation-wide evaluation of a tertiary neuroscience teaching population. We report the characteristics of the workforce and identified areas of concern for neuroscience teaching staff. Our findings could inform strategies for improving resourcing and training that support teachers of neuroscience and student learning in tertiary institutions.

## Materials and methods

We undertook an anonymous, self-administered online survey of staff employed to teach neuroscience in Australian universities. In the survey, invitation letter, and survey cover page, the term “neuroscience” was defined as “*including the structure and function of the nervous system in health and disease in humans and/or other species*”. It was specified that such teaching may be provided within a course or unit of study that specifically focuses on neuroscience, or as part of a broader course or unit of study within science, arts, engineering, medical, dentistry, nursing, or allied health degrees, or for another other tertiary qualification. The participant study inclusion criterion was that the respondent be “currently employed to teach students about the nervous system within a course or unit of study at an Australian university and/or coordinate a course or unit of study in which the nervous system is taught at an Australian university”. Responses from people who did not teach or coordinate a course or unit of study that teaches students about the nervous system at an Australian university at the time of the survey were excluded from our analysis.

### Ethics approval

The study was approved by the human research ethics committees at the University of South Australia (protocol 204168) and the University of Sydney (protocol 2021/545). The study was conducted in accordance with the Code of Ethics of the World Medical Association (Declaration of Helsinki). Informed consent was obtained from each respondent prior to participation, as described below.

### Participant recruitment

To identify eligible participants, two authors (AL, FK) searched the websites of all 43 public and private universities registered with the Australian Government Tertiary Education Quality and Standards Agency to identify courses or units of study that include neuroscience content and the staff members who teach them; searches were undertaken during 1 July ‒ 30 September 2021. Each university website was searched using a standardised approach; specifically, course/degree/program handbooks on the university website were searched for the keywords “neuro”, “neuroscience”, “brain”, and “nervous system”. For university websites without searchable handbooks, we searched the university website for the same terms, and the search results were manually assessed for course/degree/program titles and outlines. We also searched the university websites for the email addresses of the listed course coordinators or lecturers for each identified unit of study. One university (University of Canberra) did not list staff email addresses but included phone numbers; we phoned each relevant staff member at this university to ask whether they would provide their email address and agree to receive an email invitation to the study.

Some tertiary institution neuroscience teachers are not listed as teaching staff on university websites, including research fellows and doctoral students who provide some teaching in addition to their primary role or only in their area of expertise. To ensure that we included a broad cross-section of neuroscience teaching staff, identified neuroscience teachers were asked to share information about the invitation to the survey to their neuroscience teaching colleagues, including casual teaching staff (snowball sampling). We also posted advertisements of the survey on two publicly available social media sites, as described below.

### Survey design

The complete survey is included in the S1 Appendix. The first page of the online survey provided general information about the study and the participant information sheet. Two initial questions sought acknowledgement that the survey respondent had read the participant information sheet and consented to participate. Twenty-five primary questions (four with subsections) focused on teaching experience, qualifications, employment, and demographic characteristics; depending on the question, binary (yes/no), single- or multiple selection multiple choice, multiple-selection multiple choice, optional short-text responses, open-ended responses, and ordinal scale responses were possible. Adaptive questioning applied logic algorithms that displayed only relevant questions according to respondents’ previous answers. The survey also included a “back” button to enable respondents to review and change their answers. Responses were excluded for respondents who did not provide consent or for whom five or more responses to the 25 primary survey questions were missing.

Question 1—“*Are you currently employed to teach students about the nervous system (human or other species) within a course or unit of study at an Australian university or do you coordinate a course or unit of study in which the nervous system is taught at an Australian university*?” (Yes/No)—was designed to confirm eligibility for the study; respondents who answered “no” were excluded from the study. Questions 2 to 6 sought information about the types of courses/units of study and degrees/programs in which the respondent delivered neuroscience content to students (over the last five years, over entire career), how long they had taught these courses/units of study and degrees/programs, (over entire career), and their role in these courses/units of study or degree/program (over past 12 months). The subsections of Question 6 asked the respondent whether they had received or provided training for the neuroscience content from/to other staff involved in the unit/course, and about the nature of such training. Question 7 asked about the number of hours of neuroscience content delivered by the respondent over the past 12 months and the mode of content delivery. Question 8 and its subparts asked about formal supervision of students completing honours or higher degrees by research with a focus on the structure or function of the nervous system. Questions 9 and 11 asked about teaching resources in the respondent’s institution (e.g., anatomy laboratory, bespoke neuroscience teaching resources/programs) and Question 10 was about whether the survey respondent’s institution offered other opportunities for students to be involved in neuroscience research (e.g., senior year elective class, holiday research scholarship/program). Questions 12 to 15 collected information on age, gender, language spoken at home, and Indigenous status, based on the wording of questions in the 2021 Australian census [5]. Questions 16 and 17 asked about employment type and Questions 18 to 21 were about qualifications completed or in progress. Question 22 asked whether participants had completed any short courses, workshops, or other training in teacher-related skills, and Questions 23 and 24 asked what training and resources would be beneficial for teaching neuroscience. Question 25 provided an opportunity for respondents to provide any further comments they wished to make about the teaching of neuroscience.

### Survey administration

**Fig 1:**
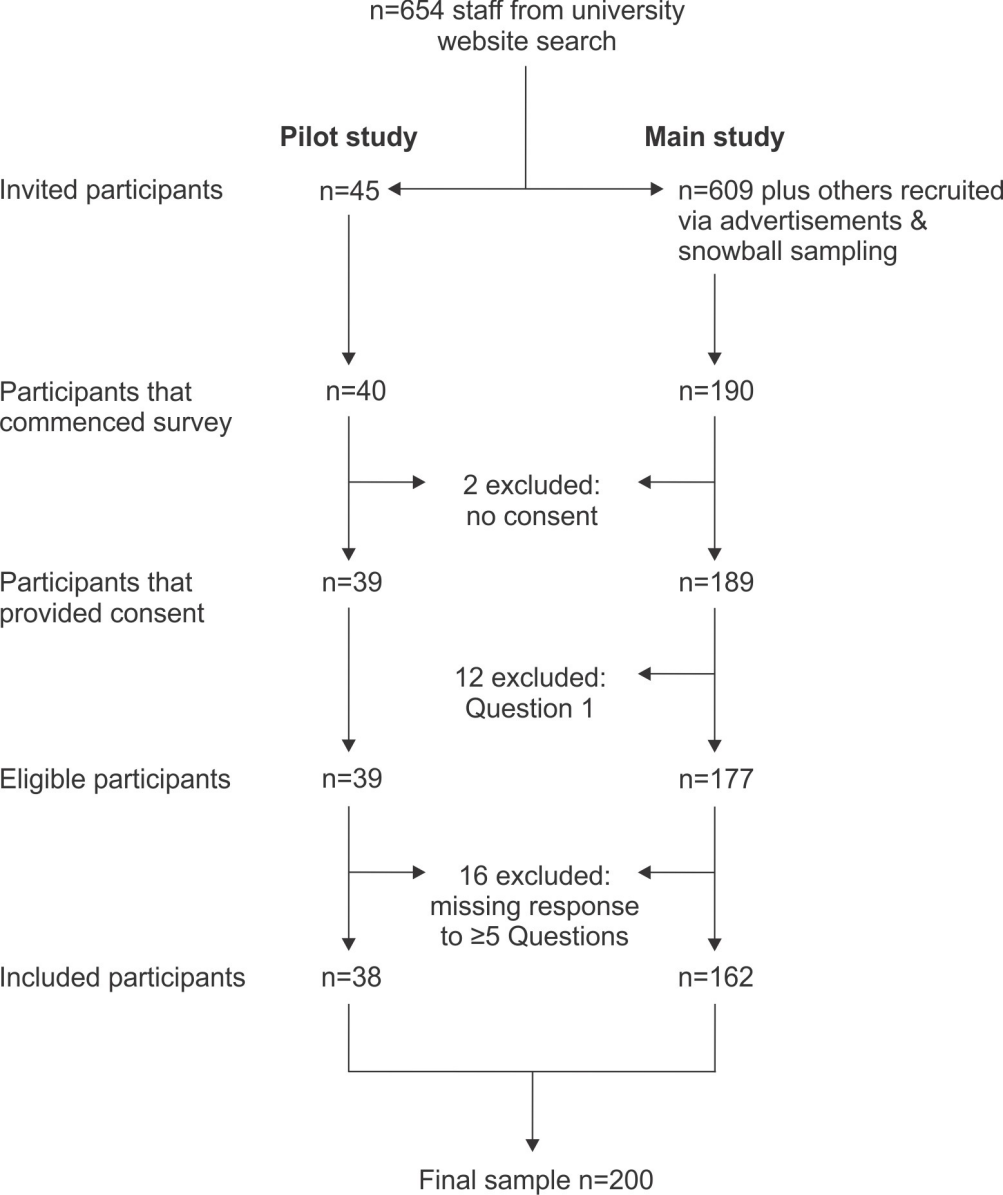
Flow chart illustrating participant recruitment, inclusion/exclusion criteria, and the final sample size.

Study data were collected and managed using REDCap electronic data capture tools (version 12.5.8), hosted at the University of Sydney. Survey usability and functionality was pre-tested by five neurosurgical health professionals not currently active in university teaching. The survey was then administered to eligible teaching staff in two waves: a pilot study and the main study. The pilot study was conducted to establish construct validity, required survey completion time, and the utility and suitability of the short-text and open-ended responses for qualitative analyses. Forty-five academic staff identified by the university website search and known to the research team were emailed invitations to participate in the pilot study on 27 September 2021; to avoid perceived coercion, the invitation indicated that participation was voluntary and that responses would be anonymous. Forty of the 45 staff commenced the pilot survey; two were excluded by the exclusion criteria described above (Fig 1).

For the main study, all other academic staff identified by the university website search were invited by email on 6 December 2021 to participate in the survey and also asked them to forward the invitation to other eligible staff. Reminder email invitations were sent on 17 January and 7 February 2022. Advertisements were also placed on the publicly available Australasian Neuroscience Society website (www.ans.org.au) and Facebook page and the Australian Women in Neuroscience Facebook page. Participants could complete the survey until midnight on 16 March 2022. We could not identify whether respondents in the pilot or main studies completed the survey more than once because IP tracking was inactivated to protect anonymity, this also did not allow us to determine the proportion of subjects who completed the survey following our targeted invitation email compared to those responding following invitation by a peer (snowball sampling).

A prize draw (for six AU$50 retail store vouchers) was offered as an honorarium for participating in the pilot or main studies. The respondent was prompted to click on a link that redirected them to a separate site that asked for contact details (name, email address, university) for the prize draw.

### Data analysis

Data were exported from REDCap to Microsoft Excel. Data quality was assessed for missing values, field validation errors, and incorrect values for calculated fields. The datasets generated and analysed during the study are available as S2 Appendix. Data likely to lead to identification of individual participants has been omitted. Graphs were created in SigmaPlot for Windows 15 (build 15.0.0.13; Inpixon). Conventional content analysis [6] was performed on free text responses to Question 24: key words and phrases were identified to generate codes, and each code was assigned a label and definition; linked or related codes were sorted into categories. The free text responses were entered into a Microsoft Excel spreadsheet with columns for each of the codes. Two investigators (GT, KD) independently coded each free text response for the presence of each code; discrepancies (six of 70 responses) were adjudicated by a third author (MC). Quantitative data were summarised as means with standard deviations (SD), medians with interquartile ranges (IQR), or proportions. Gender-based differences in categorical responses by age group (18–25, 26–35, 36–45, 46–55, over 55 years) were assessed in Fisher exact tests; the strength of the association between gender and age was quantified with Cramer’s *V* coefficient (0 = no association to 1 = perfect association). Statistical analyses were undertaken in SPSS 28.0.1.1 (IBM SPSS Statistics). *P* < 0.05 was deemed statistically significant.

## Results

The website searches identified that 34 of 43 universities offered courses or units of study that included neuroscience (total number of courses/units of study: 775). The total number of identified academic staff associated with these courses or units of study was 654.

In the main study, 190 people commenced the survey; 28 were excluded (missing consent, answered “no” to Question 1, or missing responses to five or more questions), and 162 participants were included in the main study (Fig 1). Including the 38 participants in the pilot study, the total number of eligible participants was 200 (overall response rate, 31%).

**Fig 2:**
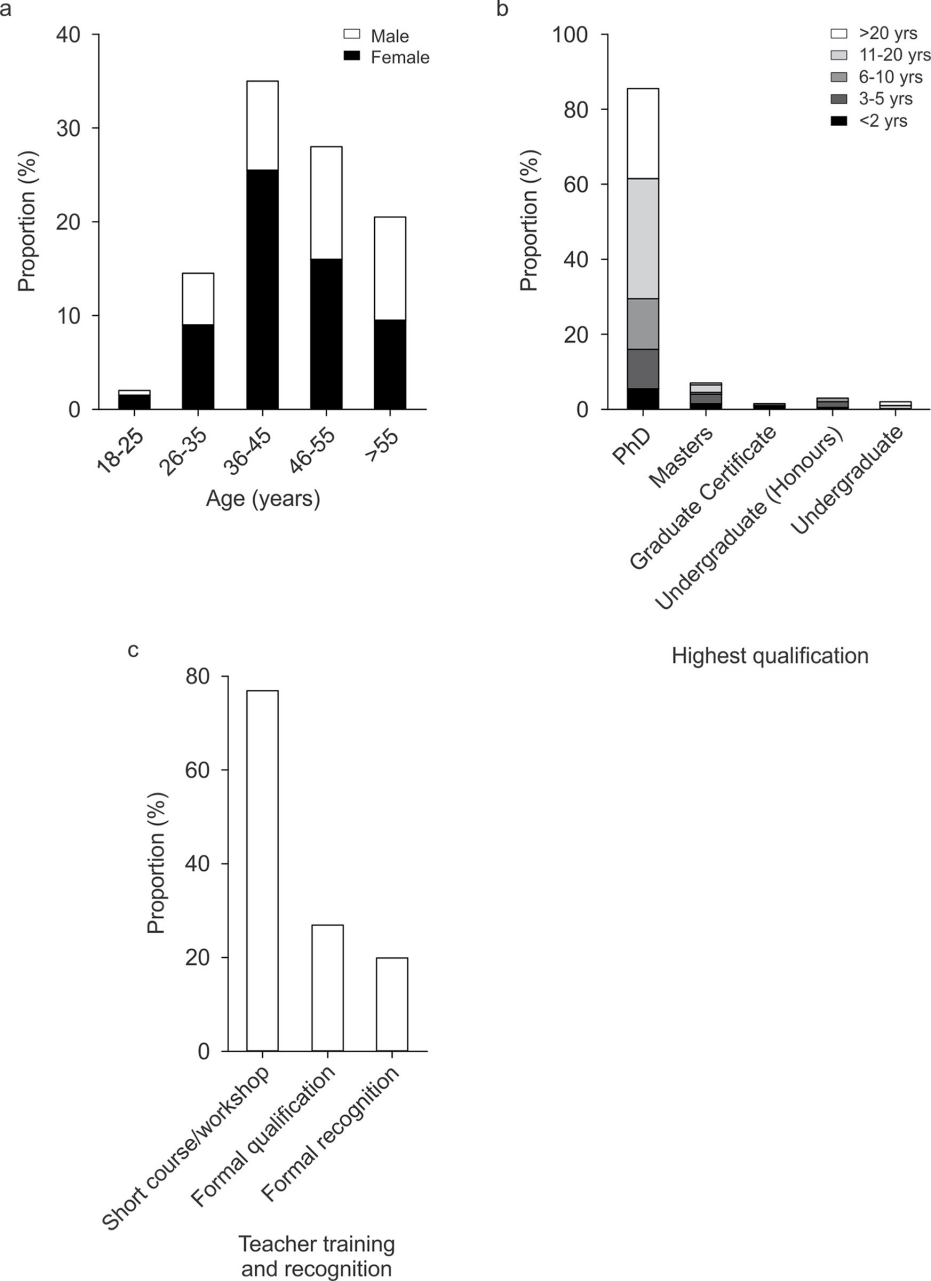
Age, gender, and qualifications of teaching staff who completed the survey. a) Age and gender (200 responses; Questions 12 and 13). b) Highest completed qualification (198 responses) and time since highest qualification was completed (Question 18). c) Formal teaching qualification at an Australian university or formal recognition of teaching expertise (200 responses; Question 21).

All participants identified as either male (38%) or female (61%; Fig 2A). The largest age group of respondents was aged 36‒45 years (35%; Fig 2A). There was a trend for an effect of gender on age (p = 0.066) but the association between gender and age was low (Cramer’s V = 0.207, p = 0.070). English was spoken at home by 81% of respondents and 19% spoke more than one language (other than English) at home. One survey respondent was an Aboriginal Australian; 199 respondents were non-Indigenous Australians.

A total of 171 respondents (86%) had completed doctoral degrees, typically 11‒20 years (32%) or more than 20 years (24%) ago (Fig 2B). The mean proportion of neuroscience or nervous system content in respondents’ highest qualification was 56±38% (median = 61%; Q1–Q3: 20–100%). A total of 153 respondents (77%) had completed short courses, workshops, or other training in teaching-related skills at their institutions, and 54 (27%) had formal teaching qualifications from an Australian university (e.g., graduate diploma of tertiary education; Fig 2C); in 32 cases, the formal teaching qualification had been completed six or more years ago. Forty respondents (20%) had received formal recognition of teaching expertise (e.g., a teaching award; Fig 2C). Twenty-three respondents (12%) were currently enrolled as students at Australian universities (15 PhD, 4 Masters, 1 Graduate Diploma, 2 Graduate Certificate, 1 Undergraduate (Honours)).

**Fig 3:**
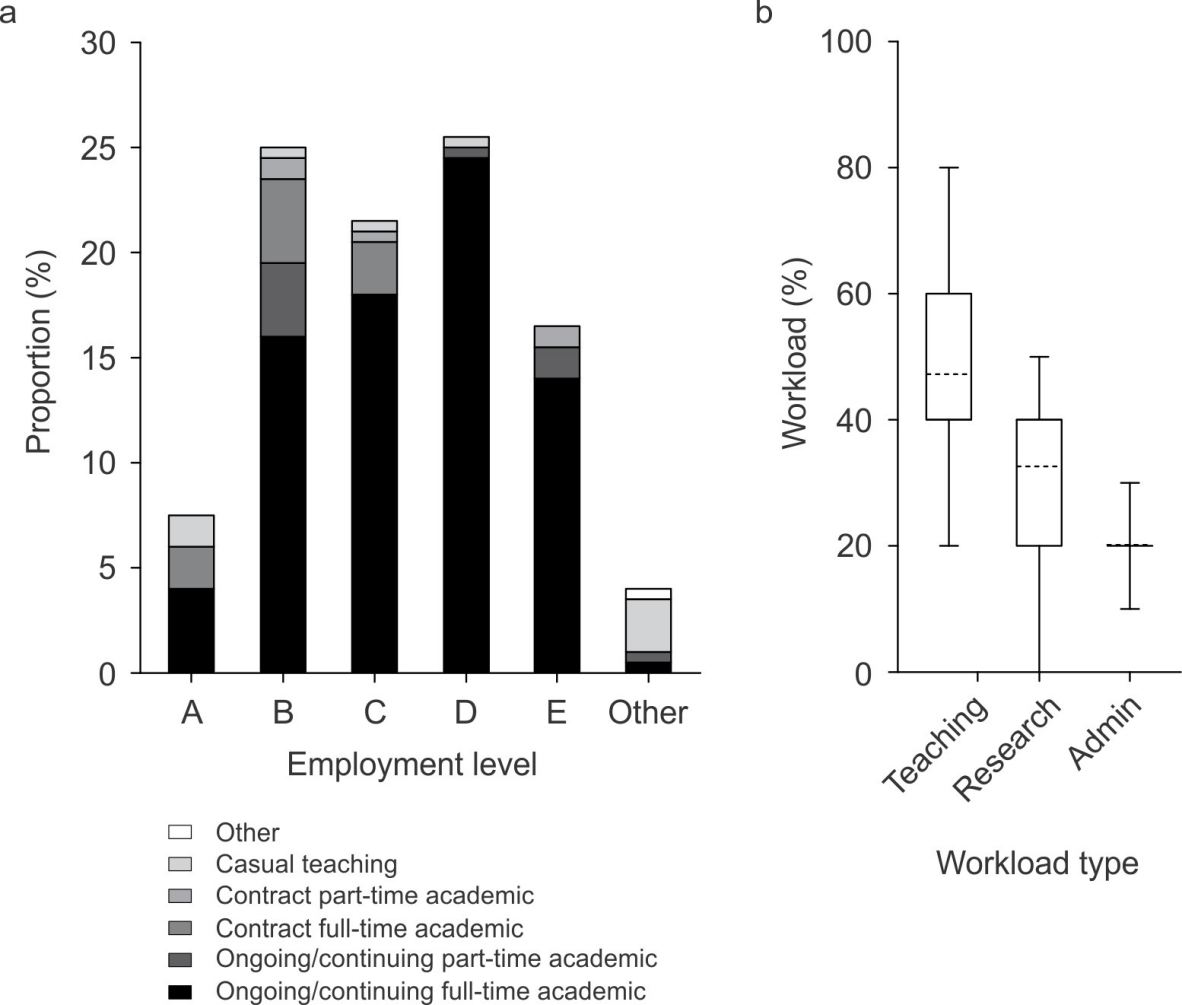
Current employment type (200 responses; Question 17). a) Type of employment and level of appointment. b) Proportions of workload allocated to teaching, research, and administration activities. The lower and upper boundaries of each box mark the 25^th^ and 75^th^ percentiles, the lower and upper whiskers the 10^th^ and 90^th^ percentiles, the dashed line the mean value.

Ninety two respondents (46%) were currently employed at Australian universities ranked globally among the top 150 universities according to the *Times Higher Education World University Rankings 2020* [7]. Most respondents had ongoing or continuing full-time academic positions (77%) and employment at Level B (Lecturer; 25%), Level C (Senior Lecturer; 22%), Level D (associate professor; 26%), or Level E (Professor; 17%) was more common than employment at Level A (Lecturer; 8%) or other appointment (4%) (Fig 3A). The respondents included more balanced teaching‒research academics and teaching-focused academics than research-focused staff (Fig 3B). Thirty three percent of the respondents reported teaching students about the nervous system in courses or units of study focused on the nervous system, 35% taught about the nervous system in courses or units on the structure and function of multiple organ systems or molecular pathways, and 32% taught students about the nervous system in both types of courses or unit of study.

**Fig 4:**
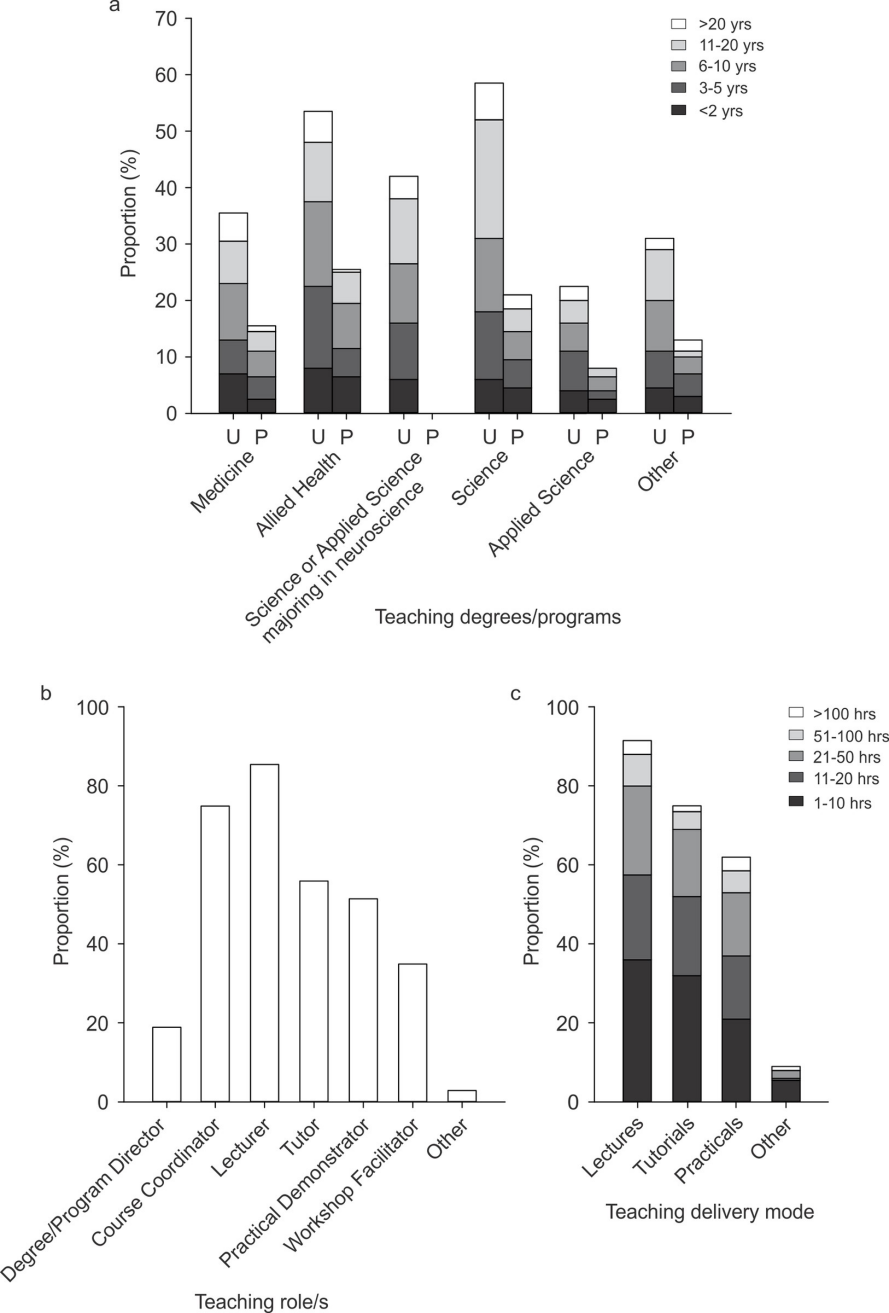
Neuroscience teaching experience (200 responses). a) Undergraduate (U; within the past 5 years) and postgraduate (P; over career) degree/program in which respondents taught students about the nervous system (Question 3). Years of experience teaching each type of degree/program is shown within each bar (Question 4 and 5). b) Type of teaching role over the past 12 months in courses/units of study or degrees/programs (Question 6). c) Time spent delivering neuroscience content in lectures (face-to-face, online, pre-recorded), tutorials or problem- or team-based learning (face-to-face or online), practical classes (face-to-face, online), or other modality (e.g., clinical setting, such a clinic or hospital ward) over the past 12 months (Question 7).

Participants were asked about the specific types of undergraduate and postgraduate degrees or programs in which they have ever taught students about the nervous system, and their number years of teaching experience in each type of degree/program (never taught, less than two years, 3‒5 years, 6‒10 years, 11‒20 years, more than 20 years). The question requested a response for all listed degrees/programs, even if the survey participant had never taught in the specified degree/program. Missing responses for this question were assumed to indicate “never taught” if the participant had selected a time range for at least one of the listed degrees/programs. Participants had more experience teaching undergraduate degrees/programs than postgraduate degrees/programs and the most frequent degrees/programs taught were in the science and allied health disciplines (Fig 4A). Fig 4B shows the survey respondents role (over the past 12 months) in the course(s)/unit(s) of study and/or degree(s)/program(s) that teach students about the nervous system. Most survey respondents performed course coordination and/or lecturer roles and facilitation of tutorials (including problem- or team-based learning approaches), practicals, and workshops were also common. The estimated number of hours spent delivering neuroscience content to students in lectures, tutorials (including problem- or team-based learning approaches), practicals, and other types of teaching (over the past 12 months) is presented in Fig 4C. Thirty two respondents (16%) received training in the content or delivery of content prior to teaching in these classes and 42% provided training to other staff. The remaining participants did not provide training (26%), were the sole teaching staff member (10%), or did not answer this question (22%). Eighty seven percent of respondents had access to anatomy laboratories for teaching purposes (79% both cadaver specimens and plastic models, 8% plastic models only). Nineteen percent of respondents reported that their institution offered bespoke neuroscience teaching resources (e.g., augmented reality presentations, customised game-based teaching, subscription to brain atlases) and the remainder of respondents reported that their institution did not offer these resources (32%) or did not know whether these resources were offered (50%).

**Fig 5:**
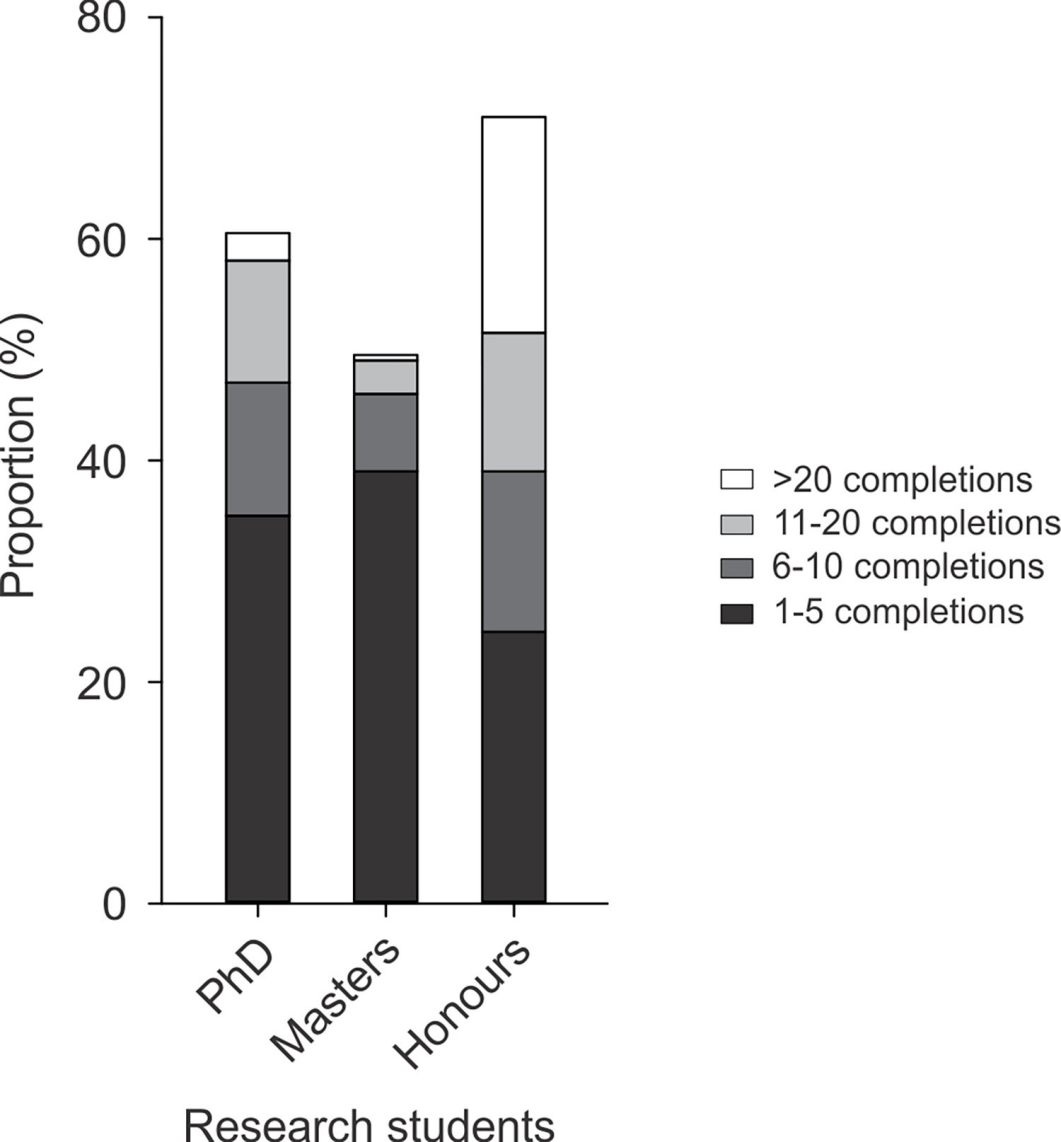
Supervision of research students (PhD, masters or honours by research) to completion as primary supervisor (Question 8). The number of student completions during the teaching staff members career is also shown within each bar.

Over the past three years, 90 respondents (45%) had been primary supervisors for students completing a Higher Degree by Research (e.g., PhD or Masters by Research) or a student completing an Honours Degree by Research with a focus on the structure or function of the nervous system, and 53 (27%) had been co-supervisors or associate/auxiliary supervisors. Most survey respondents had been a primary supervisor for at least one completed research student in their career to date, with 1‒5 completions more common than 6 or more completions (Fig 5). Sixty five percent of survey respondents said that their institution offered other opportunities for students to become involved in neuroscience research (e.g., as a senior year elective class or a holiday research program) and 14% and 22% reported no opportunities or did not know of opportunities, respectively.

Most respondents indicated they would benefit from an informal network of Australian neuroscience teaching peers (64%) and/or access to neuroscience teaching resources and/or peer-tested teaching plans/ideas (80%). The percentage of survey respondents that indicated that they would benefit from a formal mentoring system for career development in the teaching of neuroscience and/or training workshops focused on neuroscience teaching was 34% and 59%, respectively. Forty nine percent indicated that national guidelines for neuroscience teaching curricula would be beneficial. Only 6% of teaching academics indicated that none of the listed training and development opportunities would be beneficial.

The most common responses to Question 24 (“Please describe any other resources or training you feel would assist you to optimise your teaching of neuroscience”) were access to external online teaching resources (16 of 70; 23%), teacher training opportunities (9 of 70; 13%), and peer-to-peer sharing of class plans or materials (8 of 70, 11%; Table 1).

**Table 1:** Thematic analysis of 70 responses to Question 24 (“Please describe any other resources or training you feel would assist you to optimise your teaching of neuroscience”).

Category	Descriptors	Number
**Access to resources**		
External online	Brainstorm, bespoke videos, experiential learning technologies and simulation software, online database for digital 3D models, resources that highlight careers that use neuroscience skills/knowledge (authentic work-integrated learning experience), good quality resources, repository of quality digital resources, animations, customisable visual aids, interactive teaching materials, technological alternatives to tissue dissection, online lab activities, patient videos, textbooks, audiovisual resources (video tutorial), podcasts, Twitter courses/resources	16 (23%)
Infrastructure	Glass slides for microscopy, hands-on equipment for teaching psychology, access to anatomy laboratory	3 (4%)
**Peer-to-peer sharing**		
Class plans/materials	Sharing materials and ideas for practicals, tutorials, workshop, case studies, and assessment, example open-access lectures, interactive learning for students, access to question banks	8 (11%)
Help/advice	Help with practical materials and resources	2 (3%)
Mentoring	Peer mentorship from staff in higher ranked universities	1 (1%)
Network of neuroscience educators	Community of practice, shared insights with colleagues from different universities, networking and idea sharing, network of neuroscience educators who are researching and publishing in teaching and learning, website facilitating network of neuroscience teachers outside of social media platforms	5 (7%)
Pedagogy	Examples and exposure to other lecturers’ approaches to teaching	1 (1%)
**Training and professional opportunities**		
Training	Training workshops, workshops on new technologies for teaching, advanced-level courses or workshops, training in teaching an online course, training in appropriate depth of neuroscience learning/knowledge required by Allied Health students, training resources that include applied neuroscience (e.g., neuroplasticity), engineering- or applied science-focused training, undertake higher degree by research in neuroscience field,	9 (13%)
Job vacancies/staff recruitment	Advertisements of available positions	1 (1%)
**Neuroscience education research and recognition**		
Research funding	Funding for neuroscience education research	1 (1%)
Awards for excellence	Awards for neuroscience education	1 (1%)
Representation in professional bodies/societies	Satellite workshops at national conferences, more neuroscience Education representation in oral and poster presentations at national conferences	2 (3%)
**Curriculum benchmarking**	Knowing level of detail provided in other equivalent programs, national curriculum framework, shared syllabi for benchmarking	3 (4%)
**Other**		
None or don’t know	None, nil, see above, covered above, don’t know	14 (20%)
Miscellaneous		3 (4%)

## Discussion

The current data represents the first known description of a nation-wide tertiary neuroscience teaching population. The data demonstrates that Australian neuroscience teachers are primarily highly qualified, research-active teaching academics with secure employment. They teach neuroscience to students enrolled in a broad range of degrees and programs, and most have access to anatomy laboratories for teaching purposes, although access to bespoke neuroscience resources was limited.

Most jurisdictions have a quality code or standard for higher education [8, 9], and some have specific requirements regarding academic staff qualifications. For example, the 2021 Australian Government Higher Education Standards Framework [10] requires that academic teaching staff at Australian Universities have a ‘*qualification in a relevant discipline that is at least one level higher than is awarded for the course of study* (taught), *or equivalent relevant academic or professional or practice-based experience and expertise*’ [page 11, Section 3.2.3c, 10]. Exceptions to this requirement are possible (e.g., for an academic staff member who teaches a specialised component of a course, provided that they are supervised by an academic staff member who does meet the standard) [Section 3.2.4, 10]. Most survey respondents met this criterion; 86% had a doctoral qualification, and the proportion of neuroscience/nervous system content in their highest qualification was 56±38%.

Academic teaching staff in Australian universities should also have ‘*skills in contemporary teaching*, *learning and assessment principles relevant to the discipline*, *their role*, *modes of delivery and the needs of particular student cohorts*’ [page 11, Section 3.2.3b, 10]. Two lines of evidence suggest that most survey respondents met this criterion: 27% had completed university teaching qualifications, usually after completing their doctoral degree, and 77% had completed short courses, workshops, or other training in teaching-related skills at their institution. The teaching skills of one in five respondents had also been recognised by teaching awards or citations.

Academic teaching staff are also expected to have ‘*knowledge of contemporary developments in the discipline or field*, *which is informed by continuing scholarship or research or advances in practice*’ [page 11, Section 3.2.3a, 10]. Most survey respondents held balanced teaching-research positions and reported recent research activity; 45% had been primary supervisors during the past three years for students completing a Higher Degree by Research or Honours Degree by Research, with a focus on the structure or function of the nervous system.

The Australian Government Workplace Gender Equality Agency has reported that there are more female (57.8%) than male professionals (42.2%) in the Australian tertiary education sector and the proportion of ‘professionals’ with full-time, part-time, or casual employment is 43.3% (50.2% female, 49.7% male), 14.5% (72.1% female, 27.8% male), and 42.1% (57.2% female, 42.5% male), respectively [11]. However, it is unclear whether the term “professionals” in this dataset refers to academic staff or academic and administration (or other) staff. The gender distribution of our respondents was similar to that for the broader tertiary education sector but differed by employment type. Full-time employment was more typical for our neuroscience teaching staff (77% held ongoing/continuing full-time academic positions) than ‘professionals’ in the broader tertiary education sector (43.3%). Nineteen percent of respondents spoke a language other than English at home, reflecting the cultural and linguistic diversity of the Australian community (29% of the population reported speaking a language other than English at home in 2023) [12]. Less representative of the wider community, however, is the proportion of people of Aboriginal or Torres Strait Islander origin (Australian First Nations people) in the survey respondents. Only one survey respondent (0.5% of sample) identified as being of Aboriginal origin. This is lower than the proportion of people of Aboriginal or Torres Strait Islander origin in the Australian population [3.2%; 13] and the proportion of people of Aboriginal or Torres Strait Islander origin working in the Australian Higher Education sector [1.4% in 2021; 14]. Increasing the participation and employment of people of Aboriginal or Torres Strait Islander origin in tertiary education is a goal of Universities Australia, the peak body for the Australian university sector [15].

The results of our survey also provide information about the nature of neuroscience content delivery in Australian universities. Traditional methods of tertiary teaching (lectures, tutorials, practicals) still predominate in courses and units of study that include neuroscience content. Almost all respondents delivered face-to-face, online, or pre-recorded lectures, and most staff also delivered content in tutorials and practicals. The time spent providing neuroscience-related lectures, tutorials, and practicals over the past 12 months varied from fewer than 10 hours to more than 100 hours. People who reported lower content delivery time could include those in leadership or director roles, people with research fellowships, and doctoral students teaching only within their specialised area. Pleasingly, most respondents reported access to anatomy laboratories with human cadaver specimens and plastic models for teaching purposes. However, bespoke neuroscience teaching resources (e.g., augmented reality presentations, customised game-based learning, online brain atlases) were less common, mainly because institutions did not offer these resources or the respondent was unsure whether they were available. There was widespread enthusiasm (80% of respondents) for greater access to neuroscience teaching resources and peer-tested teaching plans and ideas. A range of open access resources [e.g., The Open Neuroscience Initiative: 16, 17] suitable for tertiary neuroscience teaching could be curated and disseminated through a network of neuroscience teachers. The inclusion of university-produced teaching content in such a repository would, however, be limited by intellectual property protections.

There was no clear consensus about the need for developing guidelines for neuroscience curricula; only 49% of respondents felt they would be useful. In contrasts, a major review of undergraduate biology education in the United States, involving more than 500 biologists, led to learning objectives being recommended for defined core concepts [e.g., Vision and Change in Undergraduate Biology Education: a Call to Action, American Association for the Advancement of Science: described in 18]. Lists of core concepts have also been developed for physiology [e.g. 19], biochemistry and molecular biology [20], and anatomy [21].

Our survey provides a snapshot of 200 academics who teach neuroscience in Australian universities. This data could be used by universities to benchmark resource availability and staff training and experience in neuroscience teaching. Our survey could be adapted and used in other jurisdictions to characterise the neuroscience workforce, and to identify and resolve problems at the institutional, state, and national levels, but the findings we have reported may not be directly generalisable to neuroscience teaching in other countries. Further, casual junior teachers, such as tutors and demonstrators, may not have been sufficiently represented by our respondent group, as their names and contact details are not usually publicly available on university websites; their participation in our survey relied on course coordinators extending the invitation to tutors and demonstrators.

## Conclusions

In conclusion, we undertook a survey to assess the neuroscience teaching workforce and practices in Australian universities. We found that neuroscience content is primarily delivered by highly qualified, research-active teaching academics with secure employment, but we also identified gaps in training and resources, suggesting possibilities for developing targeted approaches that would assist neuroscience teachers and improve the student learning experience.

## Supporting information

S1 Appendix Survey questions. (DOCX)

S2 Appendix Raw survey response data. (XLSX)
