# Supplementary material for: The background and experience of neuroscience teachers in Australian universities: A cross-sectional survey
Source: PLoS One. 2024 Oct 2;19(10):e0311252. doi: 10.1371/journal.pone.0311252 (PMC11446455; doi:10.1371/journal.pone.0311252)
Supplement: S1 Appendix — (DOCX) [file pone.0311252.s001.docx]

**S1Appendix.** Survey Questions

Q1. Are you currently employed to teach students about the nervous system (human or other species) within a course or unit of study at an Australian university or do you coordinate a course or unit of study in which the nervous system is taught at an Australian university?

□ yes

□ no

Q2. Do you teach students about the nervous system within a course or unit of study that:

□ focuses solely on the nervous system (e.g., a neuroscience specific course)?

□ explains the structure and function of multiple organ systems (e.g., physiology course/unit) or molecular pathways (e.g. pharmacology or biochemistry course/unit) where the nervous system forms part of a broader range of topics?

□ both of the above?

Q3. Considering the teaching you do currently or within the last five years (ie from 2016 to the present), have you taught students about the nervous system within the following UNDERGRADUATE degrees or programs? Select all that apply.

□ medical degrees (e.g., B of Medicine, B of Dentistry)

□ allied health degrees (e.g., B of Physiotherapy, B of Nursing, B of Pharmacy, B of Medical Imaging)

□ science or applied science degree majoring in neuroscience and/or the nervous system (e.g., B of Biomedical Science (Neuroscience))

□ other science degrees (e.g., B of Science, B of Medical Science, B of Biomedical Science)

□ other applied science degrees (e.g., B of Human Movement)

□ other, please specify: __________________

Q4. How many years have you taught UNDERGRADUATE students about the nervous system in the following degrees or programs? Select all that are applicable.

|  | Never taught | <2 yrs | 3-5 yrs | 6-10 yrs | 11-20 yrs | >20 yrs |
| --- | --- | --- | --- | --- | --- | --- |
| Medical degrees (e.g., B of Medicine, B of Dentistry etc) | □ | □ | □ | □ | □ | □ |
| Allied health degrees (e.g., B of Physiotherapy, B of Nursing, B of Pharmacy, B of Medical Imaging etc) | □ | □ | □ | □ | □ | □ |
| Science or applied science degree majoring in neuroscience and/or the nervous system (e.g., B of Biomedical Science (Neuroscience) etc) | □ | □ | □ | □ | □ | □ |
| Science degrees (e.g., B of Science, B of Medical Science, B of Biomedical Science etc) | □ | □ | □ | □ | □ | □ |
| Applied science degrees (e.g., B of Human Movement etc) | □ | □ | □ | □ | □ | □ |
| Other undergraduate degree | □ | □ | □ | □ | □ | □ |

Q5. How many years have you taught POSTGRADUATE students about the nervous system in the following degrees or programs? Select all that are applicable. Note this does not include supervision of Higher Degree by Research students.

|  | Never taught | <2 yrs | 3-5 yrs | 6-10 yrs | 11-20 yrs | >20 yrs |
| --- | --- | --- | --- | --- | --- | --- |
| Medical degree (e.g., Masters of Psychiatry) | □ | □ | □ | □ | □ | □ |
| Allied health degree (e.g., Masters, Graduate Certificate, Graduate Diploma) | □ | □ | □ | □ | □ | □ |
| Science degree | □ | □ | □ | □ | □ | □ |
| Applied Science degree | □ | □ | □ | □ | □ | □ |
| Other | □ | □ | □ | □ | □ | □ |

Q6. Over the past 12 months, what has been your role in the course(s)/unit(s) of study and/or degree/program(s) that teach students about the nervous system? Select all that apply.

□ degree/program director

□ course coordination

□ lectures (face-to-face, online, or pre-recorded)

□ facilitator for tutorials or problem- or team-based learning approaches (face-to-face or online)

□ facilitator for practicals (face-to-face or online)

□ facilitator for workshops (face-to-face or online)

□ other

Q6a. If Degree/program coordinator or Course coordination selected: Do you (or a person nominated by you) provide training regarding the content or delivery of the neuroscience content to other staff involved in delivering the teaching in this unit/course?

□ Yes

□ No

□ No, I am the only person teaching in this unit/course.

If yes, please describe the training provided to teachers in this unit/course (e.g., teachers attend an obligatory pre-practical training classes each week, or are provided with tutor notes outlining the key concepts to be taught, or new teachers must attend tutorial classes run by experienced tutors on that topic prior to taking the class themselves): _______________________________

Q6b. If tutorials, practicals, or workshops selected: Did you receive any training regarding the content or delivery of the neuroscience content in these classes from the degree or unit/course coordinator prior to teaching these classes?

□ Yes

□ No

□ No, I developed the content for these classes myself and thus did not require training.

If yes, please describe the training you received to prepare you to teach these classes (e.g., attended obligatory pre-practical training classes each week, were provided with tutor notes outlining the key concepts to be taught in the tutorials you lead or you were obliged or encouraged to attend tutorial classes run by experienced tutors on a specific neuroscience topic prior to leading the class yourself): ___________________________________

Q7. Approximately how many hours teaching neuroscience content in the following modes did you personally deliver to students in the past 12 months?

| Teaching mode | 0 hrs | 1-10 hrs | 11-20 hrs | 21-50 hrs | 51-99 hrs | >110 hrs |
| --- | --- | --- | --- | --- | --- | --- |
| Lectures (face-to-face, online, or pre-recorded | □ | □ | □ | □ | □ | □ |
| Tutorials or problem- or team-based learning (face-to-face or online) | □ | □ | □ | □ | □ | □ |
| Practical (face-to-face or online) | □ | □ | □ | □ | □ | □ |
| Other (e.g., teaching neuroscience in a clinical setting such as a clinic of hospital ward) | □ | □ | □ | □ | □ | □ |

Q8. During 2019‒2021, did you formally supervise a student completing a Higher Degree by Research (e.g., PhD or Masters by Research), or a student completing an Honours Degree by Research with a focus on the structure or function of the nervous system? Select all that apply.

□ yes (as Primary Supervisor)

□ yes (as Co-Supervisor or Associate/Auxiliary Supervisor)

□ no

Q8a. If yes, how many students have you supervised to completion as primary supervisor throughout your entire career? Select all that apply.

| Degree | 0 student completions | 1-5 student completions | 6-10 student completions | 11-20 student completions | >21 student completions |
| --- | --- | --- | --- | --- | --- |
| PhD | □ | □ | □ | □ | □ |
| Masters by Research | □ | □ | □ | □ | □ |
| Honours by Research | □ | □ | □ | □ | □ |

Q9. Does the institution where you currently teach students about the nervous system have an anatomy laboratory for teaching purposes?

□ yes (anatomy laboratory with cadaver specimens and plastic models)

□ yes (anatomy laboratory with plastic models only)

□ no

Q10. Does the institution where you currently teach offer opportunities for students to become involved in a neuroscience research (e.g., as a senior year elective class or a holiday research program)?

□ yes

□ no

□ I don’t know

Q11. Does your institution offer any bespoke teaching resources/programs (e.g., augmented reality presentations, customised games-based teaching, subscription to bespoke brain atlases)?

□ yes

□ no

□ I don’t know

Q11a. If yes, please describe these resources: _________________________

Please tell us about you:

Q12. Which gender do you most identify with?

□ male

□ female

□ other

Q13. What is your age group?

□ 18-25 yrs

□ 26-35 yrs

□ 36-45 yrs

□ 46-55 yrs

□ over 55 yrs

Q14. Do you speak a language other than English at home? If more than one language is spoken other than English, please indicate the language you speak most often at home.

□ no. only English

□ yes. Mandarin

□ yes. Cantonese

□ yes. Italian

□ yes. Arabic

□ yes. Greek

□ yes. Vietnamese

□ yes. other (please specify) ______________________________

Q15. Are you of Aboriginal or Torres Strait Islander origin?

□ yes. Aboriginal

□ yes. Torres Strait Islander

□ yes. Both Aboriginal and Torres Strait Islander

□ no

Q16. Is your current (primary) position of employment at an Australian university ranked in the top 150 universities according to the 2020 Times Higher Education World University Rankings? These include The Australian National University, The University of Western Australia, The University of Adelaide, The University of Melbourne, Monash University, The University of Sydney, University of New South Wales, and The University of Queensland.

□ yes

□ no

Q17. What type of employment contract do you hold in your current primary position of employment at an Australian University?

□ ongoing/continuing full-time academic position

□ ongoing/continuing part-time academic position

□ contract academic full-time position

□ contract academic part-time position

□ casual teaching position

□ other (please specify): _____________________

Q17a. What proportions of your current primary position is formally assigned to teaching, research, and administration?

| Workload | 0% | 10% | 20% | 30% | 40% | 50% | 60% | 70% | 80% | 90% | 100% |
| --- | --- | --- | --- | --- | --- | --- | --- | --- | --- | --- | --- |
| Teaching | □ | □ | □ | □ | □ | □ | □ | □ | □ | □ | □ |
| Research | □ | □ | □ | □ | □ | □ | □ | □ | □ | □ | □ |
| Administration | □ | □ | □ | □ | □ | □ | □ | □ | □ | □ | □ |

Q17b. What is your current academic/employment level?

□ Level A academic

□ Level B academic

□ Level C academic

□ Level D academic

□ Level E academic

□ Other (please specify) ____________________

Q18. When did you complete the following qualifications (select all that apply)?

| Qualification | Never completed | <2 yrs | 3-5 yrs | 6-10 yrs | 11-20 yrs | >20 yrs |
| --- | --- | --- | --- | --- | --- | --- |
| PhD | □ | □ | □ | □ | □ | □ |
| Masters Degree | □ | □ | □ | □ | □ | □ |
| Undergraduate with Honours | □ | □ | □ | □ | □ | □ |
| Undergraduate | □ | □ | □ | □ | □ | □ |

Q19. Neuroscience and/or nervous system content made up what proportion (e.g., 0%, 10%, 20%, 30%, ….. 100%) of your highest completed degree?

0% 50% 100%

|  |  |  |
| --- | --- | --- |

Q20. Are you currently enrolled as a student at an Australian university?

□ no

□ yes

Q20a. If yes, please enter the type of degree/program: _________________________

Q20b. If yes, please list the percentage (e.g., 0%, 10%, 20%, 30%, ….. 100%) of your currently enrolled degree/program that focuses on neuroscience and/or the nervous system?

0% 50% 100%

|  |  |  |
| --- | --- | --- |

Q21. Have you completed a formal teaching qualification at an Australian University (e.g., Graduate Diploma of Tertiary Education) or gained any other formal recognition of your teaching expertise (e.g., teaching awards)?

□ yes. Formal qualification

□ yes. Formal recognition

□ no

Q21a. When did you complete the formal teaching qualification?

□ <2 yrs

□ 3-5 yrs

□ 6-10 yrs

□ 11-20 yrs

□ >20 yrs

Q22. Have you completed any short courses, workshops, and/or other training on teaching-related skills at your institution?

□ yes

□ no

Q23. As a tertiary teacher of neuroscience, do you feel you would benefit from the following? Please select all that apply.

□ an informal network of Australian neuroscience teaching peers

□ a formal mentoring system for career development in the teaching of neuroscience

□ national guidelines for neuroscience teaching curricula

□ access to neuroscience teaching resources and peer-tested teaching plans/ideas

□ training workshops focused on neuroscience teaching

□ none of the above

Q24. Please describe any other resources or training you feel would assist you to optimise your teaching of neuroscience:

____________________________________________________________________________

Q25. Do you have any further comments you would like to make about the teaching of neuroscience at your institution or in Australia in general?

____________________________________________________________________________
